# CO_2_ Reforming of Biomass Gasification Tar over Ni-Fe-Based Catalysts in a DBD Plasma Reactor

**DOI:** 10.3390/molecules30051032

**Published:** 2025-02-24

**Authors:** Bianbian Gao, Guoqiang Cao, Yutong Feng, Yuting Jiao, Chunyu Li, Jiantao Zhao, Yitian Fang

**Affiliations:** 1 State Key Laboratory of Coal Conversion, Institute of Coal Chemistry, Chinese Academy of Sciences, Taiyuan 030001, China; 2 University of Chinese Academy of Sciences, Beijing 100049, China

**Keywords:** toluene, CO_2_ reforming, plasma-catalytic, Ni-Fe, syngas

## Abstract

The removal of tar and CO_2_ represents a critical challenge in the production of biomass gasification syngas, necessitating the development of advanced catalytic systems. In this study, plasma-enhanced catalytic CO_2_ reforming was employed to remove biomass tar, with toluene selected as a model compound for biomass tar. Supported Ni_x_-Fe_y_/Al_2_O_3_ catalysts, with varying Ni/Fe molar ratios (3:1, 2:1, 1:1, 1:2, and 1:3), were synthesized for the CO_2_ reforming of toluene in dielectric barrier discharge (DBD) non-thermal plasma reactors. The experiments were conducted at 250 °C and ambient pressure. The effects of various Ni/Fe molar ratios, discharge powers, and CO_2_ concentrations on DBD plasma-catalytic CO_2_ reforming of toluene to synthesis gas were analyzed. The results indicate that CO and H_2_ are the primary gaseous products of toluene decomposition, with the selectivity for these gaseous products increasing with the discharge power. Increasing discharge power leads to a higher selectivity for CO and H_2_ production. A CO_2_/C_7_H_8_ ratio of 1.5 was found to effectively enhance the catalytic performance of the system, leading to the highest toluene conversion and syngas selectivity. The selectivity of the Ni_x_-Fe_y_/Al_2_O_3_ catalysts for H_2_ and CO follows the following order: Ni_3_-Fe_1_/Al_2_O_3_ > Ni_2_-Fe_1_/Al_2_O_3_ > Ni_1_-Fe_1_/Al_2_O_3_ > Ni_1_-Fe_2_/Al_2_O_3_ > Ni_1_-Fe_3_/Al_2_O_3_. Notably, the Ni_3_-Fe_1_/Al_2_O_3_ catalyst exhibits a high CO_2_ adsorption capacity due to its strong basicity, demonstrating significant potential for both tar conversion and carbon resistance.

## 1. Introduction

The growing importance of biomass as a renewable energy source is driven by its potential to reduce dependence on fossil fuels and mitigate climate change. As a carbon-neutral resource, biomass plays a crucial role in the transition to sustainable energy systems by providing a reliable and renewable alternative for power generation, heating, and biofuel production [1]. Gasification is a critical process to utilize biomass, and during the biomass gasification process, tar formation is a significant issue due to the incomplete decomposition of organic matter, resulting in a complex mixture of hydrocarbons that condense into a liquid phase. These tars cause operational challenges, such as clogging and fouling of gasification equipment, leading to reduced efficiency and increased maintenance costs. Furthermore, tar buildup can poison catalysts in downstream processes like syngas cleaning and biofuel production, ultimately hindering the overall performance and commercial viability of biomass conversion technologies.

Biomass tar is a complex mixture of organic compounds, including polycyclic aromatic hydrocarbons (PAHs), phenols, aldehydes, and other oxygenated species [2,3]. The chemical composition of biomass tar varies depending on the feedstock and process conditions, but its high molecular weight and stability make it resistant to decomposition, causing operational issues like equipment fouling and catalyst deactivation [4]. Model compounds of biomass tar are used to investigate the reforming process because they provide a simplified, controlled environment that allows researchers to isolate and study specific tar components and their interactions during reforming. This approach helps identify optimal reaction conditions and catalyst performance before applying the findings to the complex, heterogeneous nature of actual biomass tar [5]. Regular tar model compounds that have been used include toluene, benzene, and naphthalene [6,7]. To eliminate and convert tar in fuel gas, various tar removal technologies have been developed, including mechanical separation, thermal cracking, and catalytic steam reforming. Mechanical separation is effective for removing low-concentration and simple tar compounds but suffers from low energy efficiency and is less suitable for complex tar mixtures. Thermal cracking, while effective at breaking down tar, requires substantial energy to maintain high-temperature conditions, resulting in high energy consumption and associated operational costs [8]. Moreover, the need for specialized equipment to handle the high-temperature process increases maintenance costs. Catalytic steam reforming is often considered a promising method due to its high conversion efficiency, but it is dependent on fossil fuels for energy, which leads to high energy consumption and could contribute to increased carbon emissions. In contrast, catalytic CO_2_ reforming offers a more sustainable alternative. It can utilize CO_2_ which can be sourced from renewable or waste streams, and works at lower temperatures than thermal cracking, reducing energy requirements. Additionally, it has the potential to enhance syngas yields, thus improving the overall efficiency of biomass gasification processes while also reducing harmful emissions. This makes catalytic CO_2_ reforming an attractive option for cleaner, more efficient tar removal and syngas production in biomass gasification [9,10]. CO_2_ reforming, on the other hand, offers a promising solution by utilizing CO_2_ as a reactive agent to break down tar molecules into lighter hydrocarbons, hydrogen, and carbon monoxide. This method is advantageous compared to traditional methods because it reduces CO_2_ emissions by utilizing it as a feedstock, and improves syngas production efficiency while minimizing secondary tar formation [11].

In the meantime, with the rapid development of renewable energies, renewable energy-powered electrification technologies coupled with CO_2_ reforming of biomass tar are promising alternatives to achieve efficient biomass utilization and CO_2_ valorization towards a circular economy. Plasma catalysis is an innovative technology that combines non-thermal plasma (NTP) with heterogeneous catalysis to enhance chemical reactions [12,13]. Non-thermal plasma generates highly reactive species such as electrons, ions, and radicals, which can activate and break down stable tar molecules at relatively low temperatures [14]. When coupled with a catalyst, plasma catalysis significantly improves the efficiency of the reforming process by facilitating the conversion of these reactive species into smaller hydrocarbons, hydrogen, and syngas. The synergy between plasma and catalysis allows for a lower energy input compared to traditional thermal reforming methods, as the plasma species effectively “activate” the tar molecules, while the catalyst promotes and directs these reactions [15]. This combined approach not only increases the reaction rate and selectivity but also helps maintain catalyst stability by preventing carbon deposition and surface deactivation.

Among the various types of metal-supported catalysts, Ni-based catalysts are ideal for CO_2_ reforming of toluene due to their high catalytic activity, so they have garnered significant attention in tar reforming processes [16]. However, deactivation due to coke deposition and metal sintering has limited the practical application of single-metal nickel-based catalysts in tar reforming [17,18]. To address these challenges, researchers have focused on modifying Ni-based catalysts to enhance their anti-deactivation properties. One effective approach is the introduction of a second active metal (Fe, Cu, Co, etc.) [19,20]. Studies have demonstrated that iron oxide formed by Fe after calcination can increase the lattice oxygen content of the catalyst, thereby improving its carbon resistance. In bimetallic Ni-Fe catalysts, the migration of iron oxide can effectively provide redox capacity and remove carbon deposits [21,22]. These catalysts are cost-effective and offer robust performance in high-temperature reactions, making them particularly suitable for biomass tar reforming, where efficient breakdown of complex hydrocarbons is essential. The synergy between plasma and Ni or Fe catalysts enhances tar reforming by allowing plasma-generated reactive species to interact with the catalyst surface, increasing the overall reaction rate, improving selectivity, and preventing catalyst deactivation through carbon deposition. Zou et al. prepared Fe-Ni/palygorskite catalysts for tar reforming, and found that its catalytic performance was better than that of single metal catalysts [23]. The uniform distribution of alloy on mesoporous Ni-Fe/SBA-15 results in a 90% tar conversion rate in catalytic steam reforming of tar (CSTR) [24]. Previous studies mainly focused on Ni-Fe catalyst for tar steam reforming, and its role in CO_2_ reforming was rarely reported. Considering the potential of tar CO_2_ reforming to inhibit tar formation and reduce CO_2_ content in syngas during biomass gasification, the carbon deposition resistance of bimetallic Ni-Fe catalysts and their Ni/Fe molar ratio in the tar reforming process under CO_2_ atmosphere needs to be further explored.

In this work, toluene was selected as a tar model compound and was reformed over Ni_x_-Fe_y_/Al_2_O_3_ (x, y represent the molar ratio between Ni and Fe) catalysts coupled with dielectric barrier discharge (DBD) plasma reactor. While toluene is not fully representative of all tar components in biomass, it is considered one of the key species present in the gasification products, making it a relevant model compound for this study. The effects of various Ni/Fe molar ratios (3-1, 2-1, 1-1 1-2, 1-3), discharge powers, and CO_2_ concentrations on DBD plasma-catalytic CO_2_ reforming of toluene to synthesis gas were analyzed.

## 2. Results

### 2.1. Crystalline Compositions and Texture Properties

**Figure 1:**
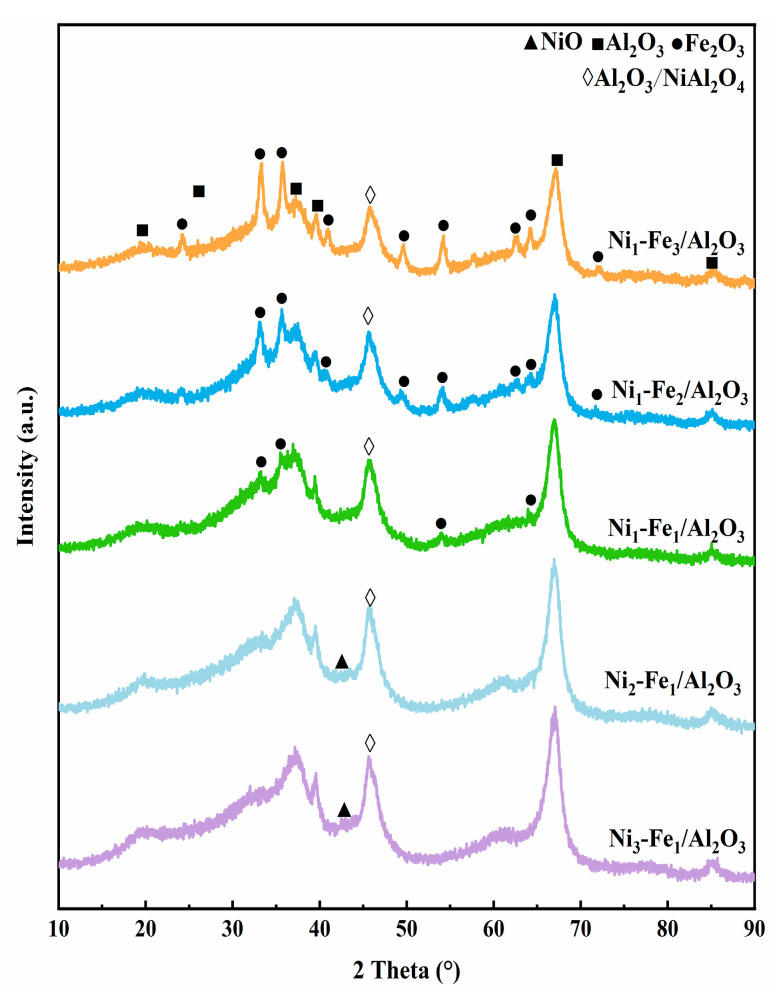
XRD patterns of Ni_x_-Fe_y_/Al_2_O_3_ catalysts.

The crystalline compositions of as-prepared Ni_x_-Fe_y_/Al_2_O_3_ catalysts were identified by X-ray diffraction (XRD), with the resulting patterns presented in Figure 1. Distinct diffraction peaks at 37.5°, 39.3°, and 66.6° are observed in several catalyst samples, corresponding to the characteristic peaks of the γ-Al_2_O_3_ support (PDF#50-0741). For the Ni_1_-Fe_3_/Al_2_O_3_ catalyst, sharp peaks associated with the Fe_2_O_3_ phase (PDF#33-0664) are present. As the Ni content increases, the characteristic peaks of metallic Ni exhibited a slight shift towards lower 2θ values, while no features corresponding to Fe_2_O_3_ were observed. This observation is likely due to the formation of NiAl_2_O_4_ species [25] and the high dispersion of the Fe phase. Notably, FeAl_2_O_4_ was not detected in the catalysts, indicating that the interaction between nickel (Ni) and Al_2_O_3_ is stronger than that between iron (Fe) and Al_2_O_3_ [26]. Among the various Ni/Fe ratio catalysts, the Ni_3_-Fe_1_/Al_2_O_3_ catalyst exhibits a slightly enhanced full width at half maximum of the diffraction lines, indicative of a higher degree of crystallinity or slightly larger Al_2_O_3_ crystallite size [27].

**Figure 2:**
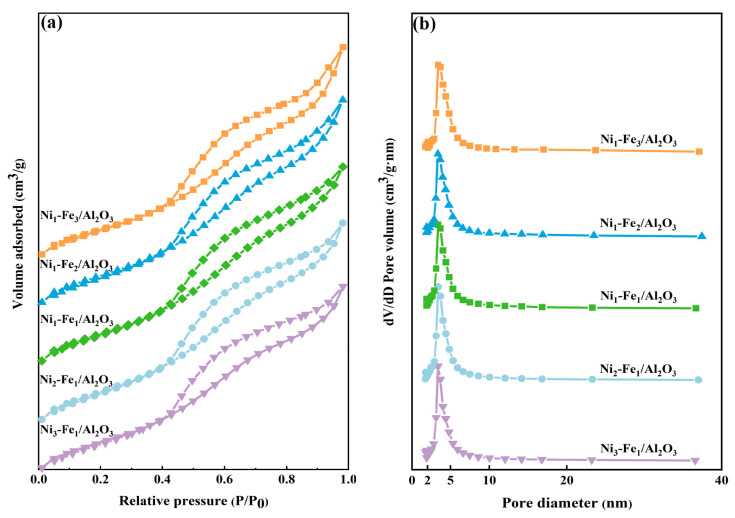
(**a**) Adsorption–desorption isotherm and (**b**) distribution of the pore diameter of Ni_x_-Fe_y_/Al_2_O_3_ catalysts.

The pore structure of Ni_x_-Fe_y_/Al_2_O_3_ catalysts significantly influences the catalytic tar reforming performance. The porosity of the as-prepared catalyst was characterized using nitrogen adsorption measurements. As illustrated in Figure 2a, all five Ni_x_-Fe_y_/Al_2_O_3_ catalysts exhibit type IV isotherms with H3 hysteresis loops, according to the IUPAC classification, irrespective of the Ni/Fe ratio [27]. This suggests that varying the Fe content does not significantly alter the mesoporous structure of the catalysts. The formation of mesoporous structures is typically governed by the synthesis conditions of the support material, such as the calcination temperature. In this case, Ni and Fe metal nanoparticles are primarily loaded onto the surface of the support or within the pores as active components, rather than directly contributing to the construction of the mesoporous framework. As a result, altering the Fe content mainly impacts the catalytic activity without disrupting the integrity of the original mesoporous structure. The specific surface area, total pore volume, and average pore size of the catalysts are summarized in Table 1. Among them, the Ni_3_-Fe_1_/Al_2_O_3_ catalyst exhibits the largest specific surface area and pore volume, attributed to the high dispersion of active metal nanoparticles on the support surface [28], which preserves the pore structure. The pore size distribution curve (Figure 2b) shows a narrow pore size distribution range (2–5 nm), further confirming the mesoporous structure of all catalysts [29]. The mesoporous structure provides ideal sites for accommodating macromolecular tar during the reforming reaction and offers numerous available channels to facilitate the diffusion and transport of tar and CO_2_ [30]. The transport path of reactants is not confined solely to the pores; however, the presence of pores enhances the likelihood of reactants interacting with the active sites, thereby improving the selectivity of the reaction.

### 2.2. Ni_x_-Fe_y_/Al_2_O_3_ Morphologies

**Figure 3:**
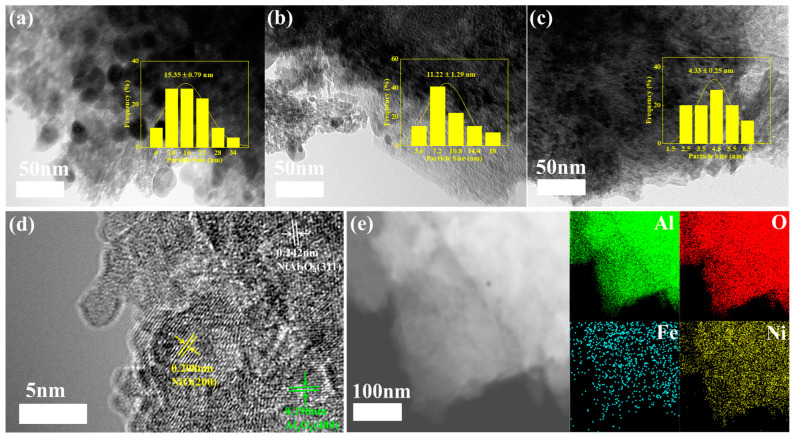
TEM images and particle size distribution of (**a**) Ni_1_-Fe_3_/Al_2_O_3_, (**b**) Ni_1_-Fe_1_/Al_2_O_3_ and (**c**) Ni_3_-Fe_1_/Al_2_O_3_, (**d**,**e**) HR-TEM images of Ni_3_-Fe_1_/Al_2_O_3_, and HAADF and EDX elemental mapping of Ni_3_-Fe_1_/Al_2_O_3_.

The surface morphology and metal dispersion of the Ni_x_-Fe_y_/Al_2_O_3_ catalysts were further analyzed using transmission electron microscopy (TEM). As shown in Figure 3a–c, the average particle sizes of the Ni_1_-Fe_3_/Al_2_O_3_, Ni_1_-Fe_1_/Al_2_O_3_, and Ni_3_-Fe_1_/Al_2_O_3_ catalysts were 15.4 nm, 11.2 nm, and 4.3 nm, respectively. Notably, the Ni_3_-Fe_1_/Al_2_O_3_ catalysts exhibit the best metal dispersion, indicating that the appropriate iron doping can significantly promote the dispersion and reduce the nanoparticle sizes. Previous studies have highlighted that catalysts containing small-sized nickel nanoparticles exhibit superior tar conversion activity and improved resistance to carbon deposition [30]. In order to further analyze the crystal structure of Ni_3_-Fe_1_/Al_2_O_3_, we use fast Fourier transform and fast inverse Fourier transform on the selected region of the HRTEM image to obtain the information of its lattice fringe space. The HR-TEM images in Figure 3d show clear lattice streaks of metal nanoparticles. The interplanar spacings of 0.208 nm and 0.242 nm correspond to the NiO (200) and NiAl_2_O_4_ (311) crystal planes, respectively. Additionally, Al_2_O_3_ (400) crystal planes were also identified, which supports the XRD results. The findings indicate that the strategic addition of Fe helps to prevent the sintering of active metal particles and further reduces the average particle size. Furthermore, high-angle annular dark-field scanning transmission electron microscopy (HAADF-STEM) image and the corresponding energy-dispersive X-ray (EDX) elemental mapping of the Ni_3_-Fe_1_/Al_2_O_3_ catalyst (Figure 3e) reveal the uniform distribution of Ni and Fe across the Al_2_O_3_ support. These results provide crucial insights into the catalyst’s structural characteristics and its potential for efficient catalytic performance.

### 2.3. Redox Properties and Basicity

**Figure 4:**
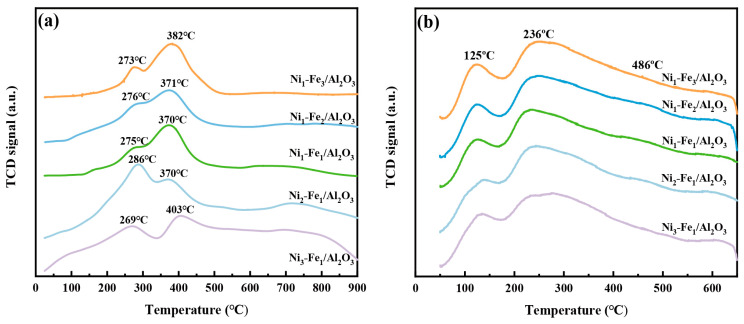
H_2_-TPR (**a**) and CO_2_-TPD (**b**) of the Ni_x_-Fe_y_/Al_2_O_3_.

Hydrogen programmed reduction (H_2_-TPR) was conducted to investigate the reducibility of metal oxides on the Ni-Fe/Al_2_O_3_ catalyst surface (Figure 4a). All samples exhibited three distinct H_2_ consumption peaks within the 200–900 °C temperature range. Specifically, the peaks around 269~286 °C are attributed to the reduction of Fe_2_O_3_ to Fe_3_O_4_ on the catalyst surface, as well as the surface reduction of nickel monoxide, which is likely influenced by its weak interaction with the Al_2_O_3_ support. The peaks around 370~403 °C are associated with the reduction of Fe_2_O_3_ to Fe_3_O_4_ within the catalyst lattice [31,32]. For the Ni_3_-Fe_1_/Al_2_O_3_ catalyst, a minor peak at 269 °C, a principal peak at 403 °C, and a broad peak centered around 750 °C was noted. The peak observed at 750 °C is associated with the reduction of NiAl_2_O_4_ to metallic Ni [33,34]. These observations are in strong agreement with the X-ray diffraction (XRD) analysis, confirming the presence of the corresponding phases. Among all the catalysts evaluated, Ni_3_-Fe_1_/Al_2_O_3_ exhibits the highest reduction temperature at 403 °C. This phenomenon can be attributed to the particularly strong metal–support interaction, which facilitates a high degree of metal dispersion on the Al_2_O_3_ support surface. The enhanced reducibility of the Ni_3_-Fe_1_/Al_2_O_3_ catalyst, as demonstrated by the H_2_-TPR results, is expected to generate a greater number of active sites, thus enhancing its catalytic activity.

In the case of CO_2_ reforming, CO_2_ acts as an acid, while the catalysts serve as bases [35]. Consequently, the basicity of catalysts significantly influences the reaction in tar CO_2_ reforming. The surface alkaline sites were characterized using CO_2_ temperature programmed desorption (CO_2_-TPD). The CO_2_ desorption peaks are associated with the interaction between acidic CO_2_ and basic sites on the catalyst surface. As illustrated in Figure 4b, desorption peaks observed in the range of 50–200 °C correspond to weakly alkaline sites, which are primarily provided by surface hydroxyl groups [1]. Stronger peaks occurring between 200 and 500 °C are associated with moderately basic sites, representing the acid–base Lewis pairs. The Lewis basic sites facilitate the adsorption and activation of CO_2_ by providing electron pairs to the carbon atom in CO_2_. In catalytic reactions, Lewis bases can stabilize cationic intermediates or activate electrophilic species. For instance, they can enhance the cleavage of C-H bonds in tar, leading to the formation of activated carbon species and hydrogen (H_2_), which serve as feedstock for subsequent reactions involving CO_2_ [36]. The high temperature peak above 500 °C is related to strongly basic sites facilitated by low coordination surface O^2−^ [37]. The notable distinction among the Ni_x_-Fe_y_/Al_2_O_3_ catalysts with varying Ni/Fe ratios lies in the desorption peak associated with medium-strong basic sites. The total number of basic sites on the Ni_x_-Fe_y_/Al_2_O_3_ catalysts increased in the following order: Ni_3_-Fe_1_/Al_2_O_3_ > Ni_2_-Fe_1_/Al_2_O_3_ > Ni_1_-Fe_1_/Al_2_O_3_ > Ni_1_-Fe_2_/Al_2_O_3_ > Ni_1_-Fe_3_/Al_2_O_3_. In general, a higher alkalinity on the catalyst surface facilitates the cleavage of C-C bonds, enhances the activation of adsorbed CO_2_, promotes the oxidation of carbon deposits at active sites, and helps reduce carbon accumulation on the catalyst surface [38]. Therefore, the Ni_3_Fe_1_/Al_2_O_3_ catalysts are expected to exhibit superior catalytic performance in toluene CO_2_ reforming.

### 2.4. Surface Chemical Properties

**Figure 5:**
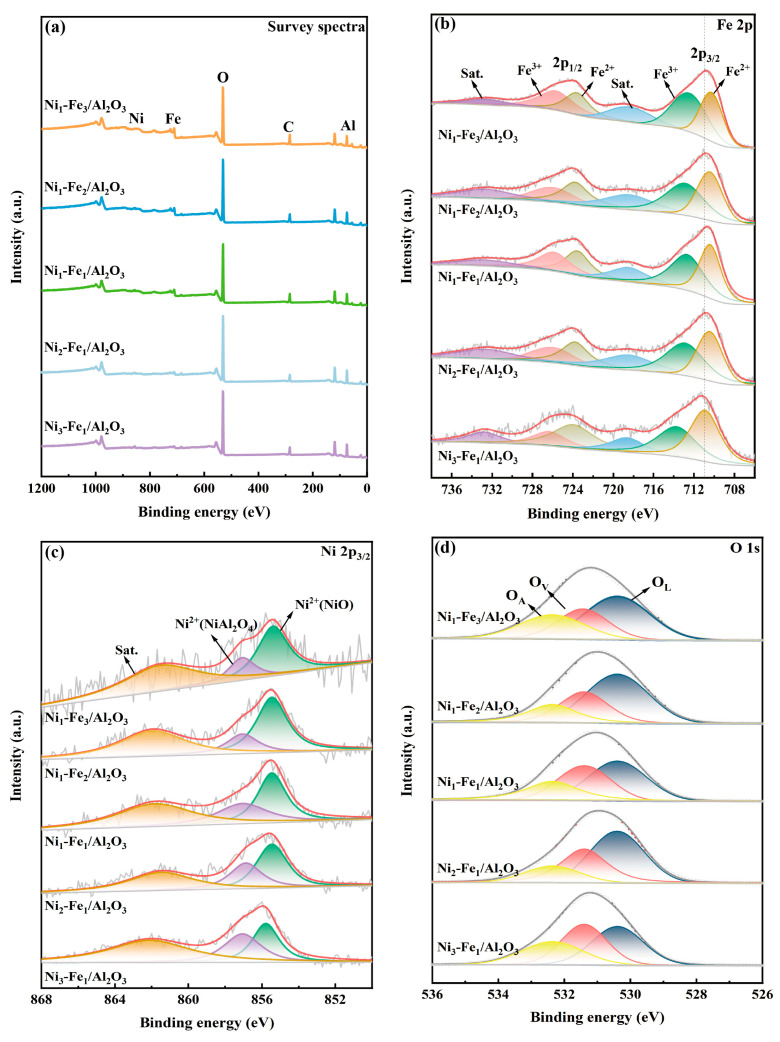
XPS spectra of Ni-Fe/Al_2_O_3_ catalysts. (**a**) The survey spectra; (**b**) Fe 2p XPS spectra; (**c**) Ni 2p3/2 XPS spectra; (**d**) O 1 s XPS spectra (O_A_ represents surface adsorbed hydroxyl oxygen, O_V_ represents defect oxygen vacancy, O_L_ represents lattice oxygen).

The electronic states and chemical composition of the elements on the surface of the Ni_x_-Fe_y_/Al_2_O_3_ catalysts were characterized using X-ray photoelectron spectroscopy (XPS). Figure 5a presents the survey spectra of Ni_x_-Fe_y_/Al_2_O_3_ catalysts, revealing that the chemical composition of the Ni_x_-Fe_y_/Al_2_O_3_ catalysts primarily consists of Ni, Fe, Al, and O. This observation is consistent with the results obtained from EDX analysis. In Figure 5b, the Fe 2p spectra can be deconvoluted into six peaks. The peaks at approximately 710.3 eV and 723.9 eV are attributed to Fe^2+^, while those at approximately 712.5 eV and 727.1 eV are attributed to Fe^3+^ [39,40]. The remaining two peaks are identified as satellite peaks [41]. As the Ni content increases, the binding energy of Fe 2p in Ni_x_-Fe_y_/Al_2_O_3_ shifts to a lower value. This observation confirms the presence of a strong electronic interaction between Ni and Fe, resulting in the presence of different Ni contents affecting the chemical environment of metal Fe. Figure 5c illustrates the high-resolution XPS spectrum of Ni 2p_3/2_, which consists of peaks corresponding to Ni^2+^ (855.5 eV) and NiAl_2_O_4_ (856.9 eV), attributed to air exposure, along with satellite peaks at 862.0 eV [33,42]. In the Ni 2p_3/2_ spectra of the catalysts, the relative abundance of Ni^2+^ species increases significantly with higher Fe content, resulting in a more pronounced presence compared to Ni^3+^. This trend is accompanied by a reduction in binding energy, indicating a stronger interaction between Ni and Fe with increasing Fe content.

In Figure 5d, the O 1s spectrum is deconvoluted into three distinct oxygen species of the metal oxide: lattice oxygen (530.4 eV), defect oxygen vacancy in hypoxic coordination (531.4 eV), and surface adsorbed hydroxyl oxygen (532.3 eV) [43,44,45]. The surface chemical states of the catalyst are further compared, and the surface element composition is listed in Table 2. It is noteworthy that on Ni_3_-Fe_1_/Al_2_O_3_ catalyst, a small amount of iron doping disrupts the orderly arrangement of nickel atoms, leading to defects within the crystal structure [36]. These defects predominantly manifest as oxygen vacancies, which account for the highest proportion of oxygen vacancies (35.4% relative peak area). The presence of oxygen vacancies induces lattice distortion, enhances the mobility of lattice oxygen, and enables it to participate in the toluene reforming reaction as active surface oxygen [46]. Furthermore, the percentage of Ni^2+^(NiAl_2_O_4_) increases significantly with decreasing Fe content, suggesting that the addition of an appropriate amount of Fe can drive more Ni into the Al_2_O_3_ lattice to form NiAl_2_O_4_, thereby enhancing the interaction between the metal and support. These findings demonstrate that an optimal amount of Fe can effectively regulate the electronic structure of the Ni-based catalysts, providing more abundant and stable active sites for toluene cracking reaction.

Based on the characterization results, the Ni_3_-Fe_1_/Al_2_O_3_ catalyst demonstrates superior catalytic properties, largely due to its large specific surface area and small particle size, which provide more active sites for the reaction and significantly improve its activity. The combination of H_2_-TPR, CO_2_-TPD, and XPS findings reveals that the Ni_3_-Fe_1_/Al_2_O_3_ catalyst exhibits remarkable redox capabilities, enhanced basicity, and an abundance of vacancy defects. These factors collectively contribute to its high catalytic performance, making it a promising catalyst for achieving high toluene conversion in reforming reactions. This assessment underscores the role of the catalyst’s structural and electronic properties, specifically its strong metal–support interaction, which supports a greater number of active sites, as well as its ability to facilitate CO_2_ activation and carbon deposition reduction. 

### 2.5. Plasma-Catalytic Acativity Evaluation

The plasma-catalytic CO_2_ reforming of toluene over Ni_x_-Fe_y_/Al_2_O_3_ catalysts with various Ni-Fe ratios was evaluated in a DBD plasma reactor.

#### 2.5.1. Effect of Discharge Power on CO_2_ Reforming of Toluene

**Figure 6:**
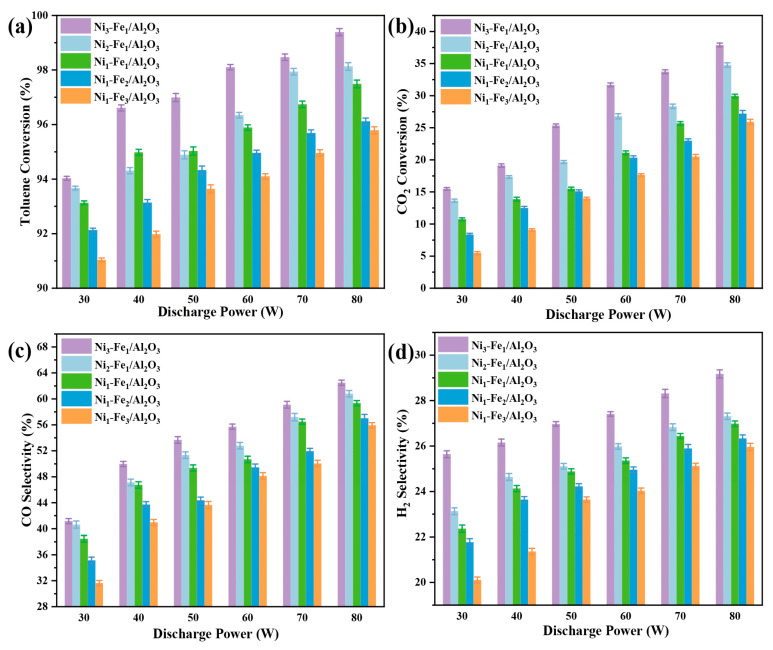
Effect of discharge power on the plasma-catalytic CO_2_ reforming performance of toluene over different Ni_x_-Fe_y_/Al_2_O_3_ catalysts: (**a**) toluene conversion, (**b**) CO_2_ conversion, (**c**) CO selectivity, and (**d**) H_2_ selectivity.

In this study, the effect of varying discharge power on toluene CO_2_ reforming catalyzed by Ni_x_-Fe_y_/Al_2_O_3_ catalysts in a DBD reactor was investigated, with the reaction temperature maintained at 250 °C and a fixed C_7_H_8_ concentration. The results show that different Ni/Fe ratios significantly impact both the conversion efficiency of toluene and the energy efficiency of the process. The catalytic performance followed the following order: Ni_3_-Fe_1_/Al_2_O_3_ > Ni_2_-Fe_1_/Al_2_O_3_ > Ni_1_-Fe_1_/Al_2_O_3_ > Ni_1_-Fe_2_/Al_2_O_3_ > Ni_1_-Fe_3_/Al_2_O_3_, with Ni_3_-Fe_1_/Al_2_O_3_ exhibiting the best performance. This superior performance can be attributed to its small particle size (nanometer scale), large specific surface area, and strong alkalinity, all of which contribute to enhanced catalytic activity. When the discharge power is increased from 30 W to 80 W, the toluene conversion and CO_2_ conversion over Ni_3_-Fe_1_/Al_2_O_3_ catalyst rise by 6% and 145%, respectively. The selectivity of major gas products, including H_2_ and CO, exhibited a similar tendency to the toluene conversion (Figure 6c,d). This phenomenon aligns with the findings reported by Xu et al. [47] regarding the use of DBD plasma for toluene reforming. In the plasma environment, the generation of high-energy electrons facilitates the cleavage of chemical bonds within tar molecules, leading to the formation of reactive species that promote the decomposition and reforming of toluene [48]. As discharge power increases, the electric field intensity in the discharge region also increases, leading to a greater abundance of high-energy electrons [49]. This intensifies micro-discharge phenomena and creates additional reaction pathways, facilitating the transformation of toluene into desired products. Thus, under suitable temperature and discharge power conditions, the conversion of toluene and the generation of gas products can be significantly enhanced.

Additionally, the conversion rate of toluene was found to be significantly higher than that of CO_2_. This phenomenon can be attributed to the plasma-catalytic system, wherein high-energy electrons excite gas molecules or break their chemical bonds, resulting in the formation of new reactive species. Toluene’s molecular structure, with C-H bonds in the methyl group and aromatic ring having lower bond energies (3.7 eV and 4.3 eV, respectively) [50] than the dissociation energy of CO_2_ (5.5 eV), makes it more susceptible to activation and bond cleavage under plasma discharge, promoting its transformation into other products.

#### 2.5.2. Effect of CO_2_/C_7_H_8_ Ratio on CO_2_ Reforming of Toluene

**Figure 7:**
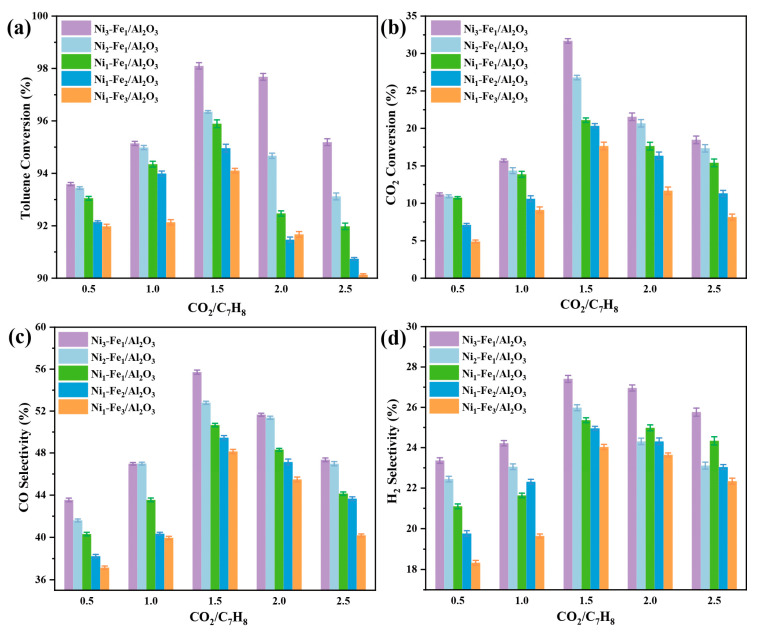
Effect of CO_2_/C_7_H_8_ ratio on the plasma-catalytic CO_2_ reforming performance of toluene over different Ni_x_-Fe_y_/Al_2_O_3_ catalysts: (**a**) toluene conversion, (**b**) CO_2_ conversion, (**c**) CO selectivity, and (**d**) H_2_ selectivity.

The reaction between CO_2_ and the carbon deposit on the catalyst surface at high temperatures plays a crucial role in maintaining the catalyst’s activity and stability. To evaluate the impact of CO_2_ concentration on toluene conversion, experiments were conducted with a fixed plasma discharge power of 60 W, while varying the molar ratio of CO_2_ to C_7_H_8_ from 0.5 to 2.5. The results show that in the CO_2_/C_7_H_8_ range of 0.5–1.5, increasing CO_2_ concentration led to higher selectivity for CO and H_2_, as illustrated in Figure 7d. This behavior can be explained by the fact that, with fixed discharge power, total gas flow rate, and reaction temperature, the number of electrons and active species in the plasma remain constant. As the CO_2_/C_7_H_8_ molar ratio increases, each toluene molecule shares more electrons and active species, resulting in a higher conversion of toluene. At a CO_2_/C_7_H_8_ molar ratio of 1.5, the maximum conversions of toluene and CO_2_ over the Ni_3_-Fe_1_/Al_2_O_3_ catalyst were achieved, reaching 98% and 32%, respectively. However, when the CO_2_/C_7_H_8_ ratio was further increased to 2.5, the conversion of toluene decreased to 95%, and the selectivity for CO and H_2_ dropped from 56% to 47% and from 27% to 25%, respectively. This indicates that excess CO_2_ input negatively affects the overall performance of the plasma-catalytic system, which is consistent with findings from other studies [51].

The decline in syngas selectivity at high CO_2_ concentration is possibly the result of a combination of the following factors. High CO_2_ concentration can promote the reverse water–gas shift (RWGS) reaction (R6) [52], which consumes H_2_ and accelerates the depletion of hydrogen in the system. As a strong oxidizing agent, CO_2_ may promote the complete oxidation of toluene (C_7_H_8_) to CO_2_ and H_2_O, rather than partial oxidation to synthesis gas. This process significantly reduces the yield of CO and H_2_. On the catalyst surface, at elevated concentrations of CO_2_, carbon dioxide may compete with toluene for adsorption, leading to the formation of stable carbonates (such as single-tooth and double-tooth carbonates), particularly at alkaline sites. These compounds obstruct the active sites and hinder the adsorption and activation of C_7_H_8_, thereby significantly reducing both conversion rates and selectivity. As an electronegative gas, CO_2_ will trap a large number of high-energy electrons at high concentrations and form negative ions. This reduces the number of available electrons for the conversion of tar, thereby affecting the formation and stability of active species within the plasma-catalytic system. Excess CO_2_ can quench active free radicals (such as H*, CH_x_*) by collision, inhibit the dehydrogenation and recombination steps of toluene, and indirectly reduce the synthesis gas generation rate.

Therefore, while moderate CO_2_ concentration enhances the conversion efficiency, excess CO_2_ interferes with the efficiency of the plasma-catalytic process. The main reason for the decrease in syngas selectivity at high CO_2_ concentration may be the combination of the enhancement of side reactions (RWGS and complete oxidation) and the covering/poisoning of the active site on the catalyst surface, and the imbalance of plasma energy distribution may exacerbate this phenomenon. A high CO_2_/C_7_H_8_ ratio is not essential for the catalytic CO_2_ reforming of toluene in a plasma-catalytic system.

The participation of active species generated by plasma discharge in surface reactions via Langmuir–Hinshelwood (L-H) or Eley–Rideal (E-R) mechanisms is considered to be a key factor in plasma–catalyst synergism [8]. Table 3 listed the possible major reactions in the plasma-catalytic toluene reforming. In the plasma environment, high-energy electrons induce the transformation of stable CO_2_ molecules into highly reactive intermediates, such as free radicals, ions, and excited molecules, through excitation, ionization, and de-excitation (R1). These intermediates are crucial for cracking and converting tar molecules. Direct electron collisions primarily dissociate CO_2_ into active species (R2) [53]. Several typical plasma-activated substances have been reported in the literature, including hydrogen radicals (·H), oxygen radicals (·O) and hydroxyl radicals (·OH) [54], as well as metastable gas molecules such as excited argon atoms (Ar*) [55]. Additionally, excited argon atoms (Ar*) collide with CO_2_, promoting its dissociation (R2), while also facilitating toluene activation through energy transfer. This process generates highly reactive intermediate products (R3–R4), which can be further decomposed through collisions with electrons or other excited species [56]. This energy transfer mechanism not only increases the reactivity of toluene molecules but also improves the overall energy utilization efficiency of the reaction system, enabling the reaction to proceed at lower temperatures. The free radicals formed from toluene decomposition (e.g., C_6_H_5_·, CH_3_·) react with oxygen species, CO, or atomic oxygen (O·) derived from CO_2_ decomposition to form oxygen-containing organic compounds such as phenols (C_6_H_5_OH) (R7–R8), aldehydes, ketones, or carboxylic acids. Additionally, atomic oxygen (O·) may oxidize toluene or its decomposition products, leading to the formation of smaller molecular products such as CO and H_2_O (R5).

#### 2.5.3. Long-Term Stability Evaluation

**Figure 8:**
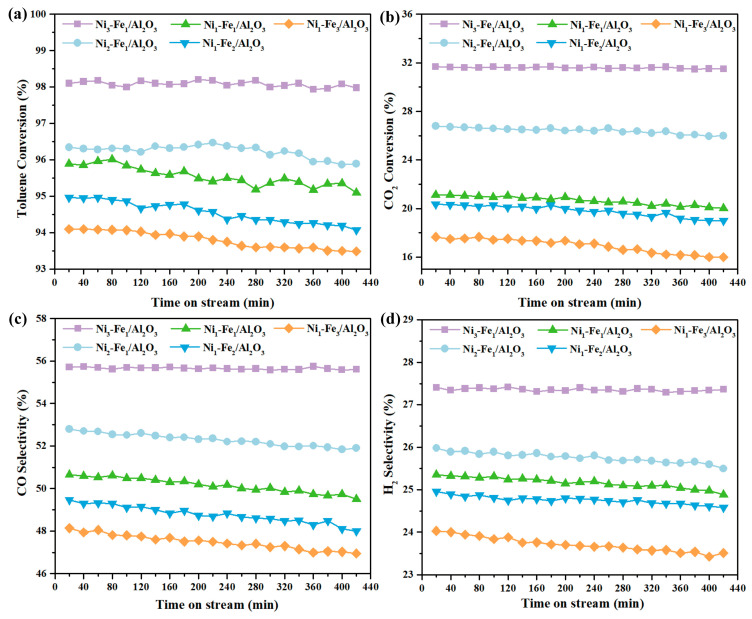
(**a**) Toluene conversion, (**b**) CO_2_ conversion, (**c**) CO selectivity, and (**d**) H_2_ selectivity as a function of time on stream over Ni_x_-Fe_y_/Al_2_O_3_ catalysts.

A long-term stability test was employed to evaluate the stability of Ni_x_-Fe_y_/Al_2_O_3_ catalysts and was conducted following the same procedure, and at a discharge power of 60 W and a CO_2_/C_7_H_8_ ratio of 1.5 over 7 h. As presented in Figure 8, the results indicate that all four indicators, including toluene conversion, CO_2_ conversion, and syngas selectivity remained stable over 7 h over Ni_x_-Fe_y_/Al_2_O_3_ catalysts. Among them, Ni_3_-Fe_1_/Al_2_O_3_ performed relatively more stably than others, its toluene conversion, CO_2_ conversion, and syngas selectivity remaining consistent with minimal variation. Moreover, a slight drop was identified over other Ni_x_-Fe_y_/Al_2_O_3_ catalysts along with time change. The long-term stability tests suggested that the as-prepared Ni_x_-Fe_y_/Al_2_O_3_ catalysts demonstrate stable characteristics toward the plasma-catalytic CO_2_ reforming of toluene.

## 3. Materials and Methods

Ni_x_-Fe_y_/Al_2_O_3_ (x, y represent the molar ratio between Ni and Fe) catalysts with various Ni-Fe ratios were synthesized via the wet impregnation method. Typically, 2 g of Al_2_O_3_ was pre-calcined at 500 °C in air for 3 h. Specific amounts of Ni(NO_3_)_2_·6H_2_O and Fe(NO_3_)_3_·9H_2_O were dissolved in 10 mL of deionized water to form precursor solutions, with a total molar mass of 0.4 mol. The precursor solutions were then mixed with 2 g of Al_2_O_3_ powder, using molar ratios of Ni to Fe of 3:1, 2:1, 1:1, 1:2, and 1:3, respectively. The precursor concentrations for each catalyst are summarized in Table 4. The mixed solution was stirred in a magnetic stirrer at 50 °C for 4 h, followed by oven drying at 110 °C overnight. The dried samples were calcined in a muffle furnace at 500 °C for 3 h with a ramping rate of 10 °C/min, then sieved to 40–60 mesh. The final catalyst samples were reduced under dielectric barrier discharge (DBD) plasma conditions for 1 h in a 10 vol% H_2_/Ar mixture (total gas flow: 100 mL·min^−1^, discharge power: 50 W). The resulting catalysts were labeled as Ni_x_-Fe_y_/Al_2_O_3_, with a total metal loading of 5 wt% and varying Ni/Fe ratios.

Elemental composition was determined using inductively coupled plasma optical emission spectroscopy (ICP-OES, Agilent 7800, Agilent Technologies, Santa Clara, CA, USA). Prior to measurement, the catalysts were digested in an aqua regia solution overnight, then diluted with deionized water to the appropriate concentration for analysis. The surface area and pore structure of the catalysts were characterized using nitrogen adsorption–desorption isotherms obtained with a Quantachrome Autosord-1. Prior to analysis, each sample was degassed at 200 °C for 2 h under high vacuum conditions. X-ray diffraction (XRD) analysis was conducted using a Rigaku Miniflex 600 diffractometer, employing Cu Kα radiation over a 2θ range of 10° to 90°. The surface chemical states of the catalysts were examined using X-ray photoelectron spectroscopy (XPS) with a Thermo Scientific K-Alpha instrument, using an Al Kα X-ray source. Binding energy calibration was performed relative to the C 1s peak at 284.8 eV, and data analysis was conducted using Thermo Scientific Advantage software (Version 5.9) and XPSPEAK41 for peak deconvolution. The basicity of the Ni_x_-Fe_y_/Al_2_O_3_ catalysts was evaluated by CO_2_ temperature-programmed desorption (CO_2_-TPD) using a JW-HX100 instrument, Beijing JWGB Instrument, Beijing, China. In a typical experiment, 100 mg of catalyst was degassed in pure He at 150 °C for 1 h, then cooled to 50 °C. The sample was exposed to a 10 vol% CO_2_/He mixture (50 mL·min^−1^) for 1 h for CO_2_ adsorption. After purging with pure He to remove weakly adsorbed CO_2_, the temperature was increased from 50 °C to 800 °C at a rate of 5 °C·min^−1^, and the CO_2_ desorption signal was recorded. The reduction behavior of the catalysts was investigated using hydrogen temperature-programmed reduction (H_2_-TPR) on a JW-HX100 instrument. A 50 mg sample was pretreated in pure He (30 mL·min^−1^) at 200 °C for 1.5 h, followed by cooling to 50 °C. A 10 vol% H_2_/Ar mixture (30 mL·min^−1^) was then introduced, and the reduction profiles were recorded as the temperature was increased from 50 °C to 650 °C at a rate of 5 °C·min^−1^ [57].

**Figure 9:**
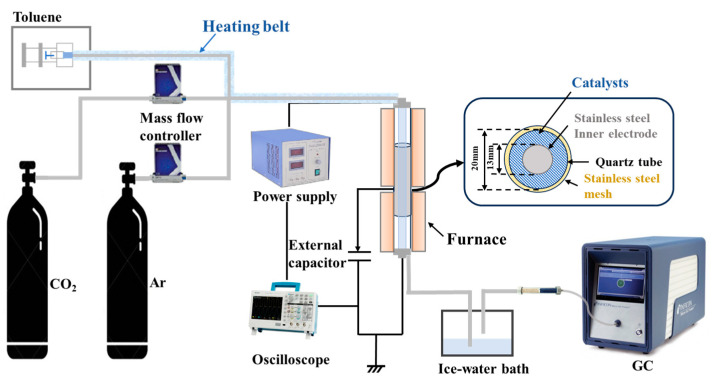
The experimental setup schematic diagram of plasma-catalytic CO_2_ reforming system.

The plasma-catalytic CO_2_ reforming of toluene was conducted in a coaxial DBD plasma reactor, as shown in Figure 9. The reactor was constructed using a quartz tube with an inner diameter of 13 mm and outer diameter of 20 mm. A mesh stainless steel wire served as the ground electrode and was wound around the outside of the quartz tube, while a high-voltage electrode was positioned inside the tube, creating a discharge gap of 3.5 mm. CO_2_ and argon were introduced into the reactor using mass flow controllers (MFCs), while toluene was injected via a high-precision syringe pump into a feed line, which was preheated to 200 °C. The reactor was maintained at 250 °C, with thermal insulation provided by a tubular furnace to minimize heat loss. A sinusoidal high-voltage power supply (Suman, Nanjing, China, CTP-2000KP) was used to generate plasma inside the reactor, with a frequency of approximately 9.7 kHz. The discharge power was determined by integrating the Q-U Lissajous diagram, and the input current and voltage were monitored using an oscilloscope (Tektronix, Beaverton, OR, USA, TBS1102C). The voltage was measured through a passive voltage probe (Tektronix, Beaverton, OR, USA, TPP0201) after capacitor voltage division (1:1000). All electrical signals were recorded for analysis. The gas phase composition was analyzed using an online gas chromatograph (Micro GC Fusion, Inficon, Zurich, Switzerland).

In each experiment, 100 mg of catalyst was placed in the discharge gap. The feed gases, including CO_2_ and toluene, were introduced into the plasma reactor in a specified ratio. The reforming reaction was carried out under the synergistic influence of the catalyst and plasma, with energy supplied by the alternating current power supply. The discharge power was calculated using the following equation:

(1) $$\mathrm{Discharge}\;\mathrm{power}\;(P)=f\;\times \;C\left(F\right)\;\times \;A(W)$$

where f represents alternating discharge frequency, A(W) is integration area in Lissajous diagram, and C(F) is the external capacitance (0.47 μF).

The conversion of toluene and *CO*_2_ and the selectivity of *CO* and *H*_2_ are defined as follows:

(2) $$x(C_{7}H_{8})=\frac{{\left[T\right]}_{in}-{\left[T\right]}_{out}}{{\left[T\right]}_{in}}\times 100\%$$

(3) $$x\left({CO}_{2}\right)=\frac{{\left[{CO}_{2}\right]}_{in}-{\left[{CO}_{2}\right]}_{out}}{{\left[{CO}_{2}\right]}_{in}}\times 100\%$$

(4) $$S(CO)=\frac{{\left[CO\right]}_{out}}{7\times {\left([T\right]}_{in}-{\left[T\right]}_{out})+{\left([{CO}_{2}\right]}_{in}-{\left[{CO}_{2}\right]}_{out})}\times 100\%$$

(5) $$S(H_{2})=\frac{{\left[H_{2}\right]}_{out}}{4\times {\left([T\right]}_{in}-{\left[T\right]}_{out})}\times 100\%$$

where [*T*]*_in_* is mole of toluene input; [*T*]*_out_* is mole of toluene output, mol; [*CO*_2_]*_in_* is mole of *CO*_2_ input, mol; [*CO*_2_]*_out_*, [*CO*]*_out_*, [*H*_2_]*_out_* are mole of *CO*_2_, *CO*, *H*_2_ in the produced gas, respectively. *S*(*CO*) and *S*(*H*_2_) are selectivity of *CO* and *H*_2_, respectively.

## 4. Conclusions

This study investigates the plasma-catalytic CO_2_ reforming of toluene, a model compound for tar, using Ni_x_-Fe_y_/Al_2_O_3_ catalysts with varying Ni/Fe ratios in a DBD (dielectric barrier discharge) plasma system. The effects of key reaction parameters—such as discharge power and the molar ratio of CO_2_ to C_7_H_8_—on toluene and CO_2_ conversion, as well as the selectivity of gas products (CO and H_2_), were systematically examined. The results indicate that increasing discharge power leads to a higher selectivity for CO and H_2_ production, which is a direct consequence of enhanced electron energy in the plasma environment. Additionally, by adding an optimal amount of CO_2_ (CO_2_/C_7_H_8_ = 1.5), the catalytic performance of the system is further improved, leading to enhanced toluene conversion and increased selectivity for synthesis gas. Among the catalysts studied, the Ni_3_-Fe_1_/Al_2_O_3_ catalyst exhibited superior catalytic activity, attributed to its unique physical and chemical properties. Specifically, the strong lattice distortion on the surface of Ni_3_-Fe_1_/Al_2_O_3_ promotes the formation of oxygen vacancies and increases its basicity, facilitating CO_2_ adsorption and activation. The study concludes that adjusting the Fe content in the Ni-Fe/Al_2_O_3_ catalysts can significantly improve their performance in plasma-catalytic CO_2_ reforming reactions. This research demonstrates the promising potential of plasma-catalytic CO_2_ reforming for biomass gasification tar removal using Ni-Fe-based catalysts. Regarding real-world applications, while the experimental results are promising, the scalability of this process requires further exploration. The integration of this method into large-scale biomass gasification systems would require addressing issues such as energy efficiency, cost-effectiveness, and long-term catalyst stability. Plasma-assisted CO_2_ reforming offers a more energy-efficient alternative to conventional tar removal techniques, such as thermal cracking and catalytic steam reforming, especially at lower operating temperatures. However, the energy consumption of the plasma system and the need for optimization to reduce plasma energy losses remain key challenges. Future work should focus on optimizing plasma reactor designs, reducing energy consumption, and improving the overall system efficiency to make this technology viable for large-scale industrial applications.

## Figures and Tables

**Table 1:** Specific surface area, pore volume, pore size and metal loading of Ni_x_-Fe_y_/Al_2_O_3_ catalysts.

Samples	S_BET_ ^a^ (m^2^·g^−1^)	Total Pore Volume ^a^ (cm^3^·g^−1^)	Average Pore Size ^a^ (nm)
Ni_3_-Fe_1_/Al_2_O_3_	160.1 ± 0.2	0.24 ± 0.03	4.8 ± 0.2
Ni_2_-Fe_1_/Al_2_O_3_	149.1 ± 0.1	0.22 ± 0.04	4.8 ± 0.3
Ni_1_-Fe_1_/Al_2_O_3_	152.9 ± 0.2	0.24 ± 0.02	4.9 ± 0.1
Ni_1_-Fe_2_/Al_2_O_3_	148.8 ± 0.3	0.24 ± 0.03	5.1 ± 0.4
Ni_1_-Fe_3_/Al_2_O_3_	147.5 ± 0.1	0.25 ± 0.05	5.4 ± 0.2

^a^ The surface area is determined by the BET equation. In addition, total pore volume and average pore size were obtained from BJH desorption data.

**Table 2:** Surface element compositions of Ni_x_-Fe_y_/Al_2_O_3_ catalysts determined by XPS spectra.

Catalysts	Surface Nickel Species		Surface Iron Species		Surface Oxygen Species		
	Ni^2+^	Ni^2+^(NiAl_2_O_4_)	Fe^2+^	Fe^3+^	O_L_	O_V_	O_A_
	Area (%)	Area (%)	Area (%)	Area (%)	Area (%)	Area (%)	Area (%)
Ni_3_-Fe_1_/Al_2_O_3_	50.4	49.6	59.9	40.1	37.8	35.4	26.8
Ni_2_-Fe_1_/Al_2_O_3_	60.2	39.8	49.8	50.2	49.1	34.6	16.3
Ni_1_-Fe_1_/Al_2_O_3_	58.0	42.0	48.2	51.8	41.6	33.3	25.1
Ni_1_-Fe_2_/Al_2_O_3_	73.4	26.6	55.6	44.4	51.8	28.7	19.5
Ni_1_-Fe_3_/Al_2_O_3_	72.2	27.8	44.2	55.8	46.2	27.0	26.8

**Table 3:** Possible major reactions in the plasma-catalytic CO_2_ reforming of toluene.

Chemical Reaction	Serial Number
Ar + e → Ar* + e	(R1)
CO_2_ + e/Ar* → CO + O· + e/Ar*	(R2)
C_6_H_5_CH_3_ + e/Ar* → C_6_H_5_· + CH_3_· + e/Ar*	(R3)
C_6_H_5_CH_3_ + e/Ar* → C_6_H_5_CH_2_· + H· + e/Ar*	(R4)
C_6_H_5_CH_3_ + O· → CO + H_2_ + LHC	(R5)
CO_2_ + H_2_ → CO + H_2_O	(R6)
C_6_H_5_· + O· → C_6_H_5_O·	(R7)
C_6_H_5_O· + H· → C_6_H_5_OH	(R8)

**Table 4:** Precursor solution concentrations used in the preparation of Ni_x_-Fe_y_/Al_2_O_3_ catalysts with various Ni/Fe ratios.

Samples	Precursor Concentration (mol/L)				
	Ni_3_-Fe_1_/Al_2_O_3_	Ni_2_-Fe_1_/Al_2_O_3_	Ni_1_-Fe_1_/Al_2_O_3_	Ni_1_-Fe_2_/Al_2_O_3_	Ni_1_-Fe_3_/Al_2_O_3_
Ni(NO_3_)_2_·6H_2_O	0.287	0.256	0.194	0.130	0.098
Fe(NO_3_)_3_·9H_2_O	0.096	0.128	0.194	0.261	0.295

## Data Availability

Dataset available on request from the authors.
